# Bacterial and fungal composition and exometabolites control the development and persistence of soil water repellency

**DOI:** 10.1093/ismeco/ycaf084

**Published:** 2025-05-20

**Authors:** Emily N Boak, Benjamin P Bowen, Katherine B Louie, Trent R Northen, Marie E Kroeger

**Affiliations:** Microbial and Biome Sciences Group, Bioscience Division, Los Alamos National Laboratory, Los Alamos, NM 87545, United States; Genomics and Systems Biology, Lawrence Berkeley National Laboratory, Berkeley, CA 94720, United States; Department of Energy Joint Genome Institute, Lawrence Berkeley National Laboratory, Berkeley, CA 94720, United States; Department of Energy Joint Genome Institute, Lawrence Berkeley National Laboratory, Berkeley, CA 94720, United States; Genomics and Systems Biology, Lawrence Berkeley National Laboratory, Berkeley, CA 94720, United States; Department of Energy Joint Genome Institute, Lawrence Berkeley National Laboratory, Berkeley, CA 94720, United States; Microbial and Biome Sciences Group, Bioscience Division, Los Alamos National Laboratory, Los Alamos, NM 87545, United States; In-Pipe Technology, Wood Dale, IL 60191, United States

**Keywords:** soil water repellency, metagenomics, metabolomics, model ecosystem, soil microbiology

## Abstract

Soil water repellency (SWR), the reduced affinity of soil for water, is a phenomenon that affects soils globally. With worsening climate change, SWR is expected to increase emphasizing the need to understand the mechanisms driving SWR development and persistence. The importance of the soil microbes in SWR has been postulated for decades, but limited research has been conducted into whole-community interactions and the role of community metabolic activity. To address this gap in knowledge, we investigated the direct effect of microbial community composition, activity, and diversity, as well as their associated metabolites on the development and persistence of SWR by inoculating microcosms containing model soils with 15 different microbial communities and quantified respiration and SWR over time. Six communities that consistently produced either a hydrophobic or hydrophilic phenotype were characterized using metagenomics and metabolomics to determine the impact of microbial and metabolite composition and diversity on SWR. We identified several bacterial genera with significant changes in abundance between SWR phenotypes including *Nocardiopsis* and *Kocuria* in hydrophilic and *Streptomyces* and *Cutibacterium* in hydrophobic. We discovered that hydrophilic communities were more positively connected when compared to hydrophobic communities, which could be due to an increase in defense mechanism genes. Additionally, we identified specific metabolites associated with hydrophilic and hydrophobic phenotypes including an increase in the osmolyte ectoine in hydrophilic and an increase in plant-derived decomposition products in hydrophobic communities. Finally, our research suggests that fungi, previously thought to cause hydrophobicity, may actually contribute to hydrophilicity through their preferential consumption of hydrophobic compounds.

## Introduction

Soil water repellency (SWR) is a physical phenomenon described as a reduced affinity of soil to water that occurs globally [1–3]. Depending on the length of time that soils resist wetting, the characterization can range from hydrophilic (i.e. water rapidly infiltrates the soil) to extremely hydrophobic (i.e. water does not infiltrate). This resistance of soil to wetting can be highly dynamic, only lasting a few seconds to hours, or more long-lasting with weeks or longer of decreased water infiltration. Hydrophobicity impacts many aspects of soil physiology and has both good and bad effects. Some benefits include soil aggregate stabilization, stabilization of soil organic matter (SOM), and prevent evaporative loss [4–6]. Hydrophobic soils can also have profound negative impacts on plant productivity such as delayed and reduced germination, decreased crop yields, lower vegetative establishment, and overall loss of fertile topsoil from erosion that costs billions of dollars annually [7–14]. Furthermore, hydrophobic soils are a major health and infrastructure liability from mudslides that cost >$1 billion annually in damage and result in fatalities [13, 15–18]. Given the extremely diverse impacts and health risks, it is critical to determine the factors controlling SWR and the development and persistence of hydrophobic soils. By understanding the major influences on SWR, we have the opportunity to control the phenotype to either induce hydrophobicity for stability or mitigate it in natural and agricultural environments for improved production and safety.

There have been many factors identified as affecting to some degree the development of hydrophobic soils including both biotic factors, such as plant root exudates and decomposition products, microbial communities, along with abiotic factors like soil temperature, texture, and clay content [13, 19–21]. However, given that SWR is a global phenomenon occurring across all soil types and varying environmental conditions, many have focused on researching biotic factors. Bond [27] suggested that repellency in soils under a range of plant species may differ due to differing suites of microorganisms associated with the plants. Other studies have shown that the presence of microorganisms alone does not fully explain SWR, and that it could be fungal species dependent [22]. Beyond the presence of microbes, microbial activity and metabolite production could be a determining factor in SWR development. A study on biological soil crusts showed that exopolysaccharides secreted by cyanobacteria prevent water penetration into the soil below [23]. Other studies have shown a correlation between microbial activity and increases in SWR [21, 24], and while several of these have suggested that microbial metabolites could have an effect on SWR, direct influences of these metabolites could not be determined [25, 26]. Although it is clear from decades of research that microorganisms are critical to the development of SWR, information on the direct impact of microorganisms on the development and persistence of SWR is extremely limited compared to other factors, with a wide-range of hypotheses from the presence of basidiomycete and arbuscular mycorrhizal fungi, changes in bacterial and fungal community composition, products produced by either microbes or plant roots, decomposing plant matter, and interactions between microbial communities [17, 21, 22, 25].

The development of SWR has been linked to soil particles becoming coated in hydrophobic organic compounds derived from either plant decomposition products or microbial metabolites [27–29]. Since microorganisms are a main driver of plant decomposition and recent research discovered that different microbial community structures (i.e. composition, abundance, diversity) can affect the decomposition products and microbial metabolites [30], it is critical to identify the effect of microbial community composition and activity on the development and persistence of SWR. This knowledge can inform land management strategies to promote microbial communities that increase water infiltration and decrease erosion. To our knowledge, no study has directly assessed the effect of microbial community composition on the development and persistence of SWR. Here, we report that specific microbial taxa and microbial metabolites are driving the development and persistence of SWR using model soil microcosms coupled with metagenomics and metabolomics.

## Materials and methods

### Soil and microcosm preparation

Microcosms were created in 125 ml serum bottles (Wheaton, Millville, NJ, USA) that contained 3.3 g of bentonite clay (Millipore Sigma, St. Louis, MO, USA), 6.6 g of washed sand (Quikrete, Atlanta, GA, USA), and 0.2 g of dried and ground blue grama grass litter to make a mock soil. This mock soil consists of ~30% clay and ~ 70% sand indicative of sandy clay loam soil. This soil texture that is naturally hydrophilic was chosen to ensure the development and persistence of SWR by microorganisms could be observed without convoluting abiotic factors. The mock soil components were mixed and autoclaved 3 times with 8–12 h between each sterilization. The mock soil (10.2 g total) was not compacted at all after mixing and allowed to settle into the serum bottle (54 mm diameter) naturally. In total, 192 microcosms were used in this experiment.

### Microbial communities

Soil microbial communities were selected as inoculum into the mock soil microcosms based off the soil’s natural water repellency. We screened soils from our soil collection [31] using a soil water drop penetration test (WDPT; as described in detail below) and selected 15 different soils to ensure we had a range of SWR status from hydrophobic to hydrophilic that would improve the likelihood that [1] the microbial communities were different and [2] that those communities would likely produce different SWR phenotypes (Supplemental Table S1).

The soil microbial community inocula were prepared by weighing 1 g of each selected soil community into separate 15 ml falcon tubes with 9 ml of sterile 1X PBS buffer to create a 1:10 dilution. These tubes were vortexed for 30 s to distribute the soil, and the solid particles were allowed to settle for 5 min. Then, 5 ml of supernatant from each community tube were transferred to 50 ml falcon tubes containing 45 ml of sterile 1X PBS buffer to make a 1:100 dilution. After thoroughly mixing, 2 ml of each inoculum was added to each respective microcosm and 2 ml of sterile 1X PBS buffer was added to the negative controls. The microcosms were then sealed with crimp caps and kept in the dark at 25°C. Given the inoculum of 2 ml into 10 g of model soil and the microcosm being sealed, the expected soil moisture content for the duration of the experiment was 20%. An aliquot of the original 1:100 soil microbial community dilution was stored at –80C for DNA extraction. For each of the 15 soils and negative controls, we had 12 replicates that allowed for destructive sampling of 3 replicates per timepoint (Days 30, 45, 60, 89).

### Carbon dioxide measurements

On Days 3, 6, 9, 14, 30, 45, 60, 75, and 89 CO_2_ concentration was measured by gas chromatography using an Agilent Technologies 490 Micro GC (Santa Clara, CA, USA) and after each measurement the microcosms had the headspace air evacuated and replaced with sterile-filtered air via vacuum pump as referenced in [32]. Cumulative respiration over the course of the experiment was used to assess overall microbial activity.

### Water drop penetration test

To measure the development and persistence of SWR, 3 replicates for each microbial community at Days 30, 45, 60, and 90 were destructively sampled and a soil WDPT was performed as described previously by the Department of Sustainable Natural Resources, altered from King, 1981 and Roberts and Carbon, 1971 [11, 33]. Briefly, 25 μl of sterilized ddH2O was dropped from a height of 1.5 cm on to the surface of the inoculated soils and the amount of time the droplet remained on the surface of the soil was measured. Based on these times, the soils were categorized into 5 levels of repellency status: not significant (<1 s), very low water repellence (1–10s), low water repellence (10–50s), moderate water repellence (50–260 s), and moderate to severe water repellence (>260 s). Using these results, 3 communities consistently categorized as hydrophilic phenotype (either not significant, very low, or low water repellency) and 3 communities consistently categorized as hydrophobic phenotype (moderate to severe water repellency) across the 3 timepoints were chosen for further metagenomic and metabolomic analysis (Supplemental Table S1). These communities were down selected for metagenomic and metabolomic analyses since our goal was to understand what microbial features and activity drive the development and persistence of soil hydrophobicity or hydrophilicity.

Following the WDPT, 3 subsamples of 2 g each from each microcosm were taken. All subsamples were immediately frozen at −80°C with subsample 1 later being used for DNA extraction, subsample 2 for non-polar exometabolites, and subsample 3 for polar exometabolites.

### Deoxyribonucleic acid extraction and sequencing

The DNA of the microbial communities was extracted from the microcosm soils using the Qiagen DNeasy PowerSoil Pro Kit™ Protocol (Qiagen, Hilden, Germany) with some modifications. Briefly, samples were vortexed in PowerBead Tube for 20 min and all centrifugation steps were run for 1.5 min. To increase DNA concentrations, a final elution volume of 30 μl was used and replicates of the same sample were combined onto one filter. Final concentrations were determined using a Qubit™ dsDNA Quantification Assay Kit (Invitrogen, Waltham, MA, USA). Extracted DNA was sent to the DOE Joint Genome Institute (JGI) for metagenomic library prep and DNA sequencing. Metagenome data assembly, structural, functional, and taxonomic annotation, and binning of metagenomic data was performed by the JGI Metagenome Workflow as referenced in Clum et al., 2021 [34]. Briefly, an Illumina fastq file is generated and processed by the JGI workflow. These results can then be integrated into the Integrated Microbial Genomes and Microbiomes (IMG/M) v 5.5 (https://img.jgi.doe.gov/) system and compared to the other stored, publicly available metagenomes for identification and characterization (Supplemental Table S2).

### Metabolite extraction

Metabolites were extracted from 2 g soil sample size. To extract polar metabolites, 1.2 ml LC–MS water was added to soil, vortexed, then sonicated in an iced water bath for 10 min. Samples were then centrifuged 5 min at 5000 rpm, vortexed again, sonicated another 4 min in an iced water bath and centrifuged (10 min at 8000 rpm). Supernatant containing extracted polar metabolites was then transferred to a new 2 ml Eppendorf, frozen and lyophilized dry (FreeZone 2.5 Plus, Labconco). To extract nonpolar metabolites, the same procedure was followed but instead of water, 1 ml of 100% MeOH added to the soil followed by vortex, sonication and centrifugation steps. Supernatant containing extracted nonpolar metabolites was then transferred to a new 2 ml Eppendorf and dried in a SpeedVac (SPD111V, Thermo Scientific). All dried extracts were stored at -80 degrees prior to LC–MS analysis.

### Liquid chromatography tandem mass spectrometry

To detect and analyze metabolites, liquid chromatography tandem mass spectrometry (LC–MS/MS) was performed on soil extracts using both normal and reverse phase chromatography. Chromatography was performed using an Agilent 1290 LC stack, with MS and MS/MS data collected using a Q Exactive or Q Exactive HF Orbitrap MS (Thermo Scientific, San Jose, CA). In preparation for LC–MS, extracted polar and nonpolar metabolites were resuspended in 120 μl of 100% MeOH containing isotopically labeled internal standards (5–50 μM of 13C,15 N Cell Free Amino Acid Mixture, #767964, Sigma; 1 ug/ml 2-amino-3-bromo-5-methylbenzoic acid, ABMBA, #R435902, Sigma), centrifuge-filtered (0.22 μM hydrophilic PVDF membrane, #UFC30GV00, Millipore), then transferred to a glass LC–MS vial.

To detect polar metabolites, resuspended water extracts were run on LC–MS using normal phase chromatography. Here, full MS spectra were collected on a QExactive mass spectrometer from m/z 70–1050 at 70000 resolution in both positive and negative mode, with MS/MS fragmentation data acquired using stepped 10, 20 and 40 eV collision energies at 17500 resolution. Chromatography was performed using a HILIC column (Agilent InfinityLab Poroshell 120 HILIC-Z, 2.1 × 150 mm, 2.7 μm, #673775–924) at a flow rate of 0.45 ml/min with a 3 μl injection volume, with HILIC column at 40°C and equilibrated with 100% buffer B (95:5 ACN:H_2_O with 5 mM ammonium acetate) for 1 min, diluting buffer B down to 89% with buffer A (100% H_2_O with 5 mM ammonium acetate and 5 μm methylenediphosphonic acid) over 10 min, down to 70% B over 4.75 min, then down to 20% B over 0.5 min, followed by isocratic elution in 80% buffer A for 2.25 min.

To detect nonpolar metabolites, resuspended MeOH extracts were run on LC–MS using reverse phase chromatography. Here, full MS spectra was collected on a QExactive HF mass spectrometer from *m/z* 80–1200 at 60 000 resolution in both positive and negative ionization mode, with MS/MS fragmentation data acquired using stepped 10, 20, and 40 eV collision energies at 15 000 resolution. Chromatography was performed using a C18 column (Agilent ZORBAX Eclipse Plus C18, Rapid Resolution HD, 2.1 × 50 mm, 1.8 μm) at a flow rate of 0.4 ml/min with a 3 μl injection volume. To detect metabolites, samples were run on the C18 column at 60°C equilibrated with 100% buffer A (100% H2O with 0.1% formic acid) for 1 min, diluting buffer A down to 0% with buffer B (100% ACN with 0.1% formic acid) over 7 min, and isocratic elution for 1.5 min, followed by column re-equilibration by returning to 100% A over 1 min and isocratic elution for 1 min.

Samples consisted of three biological replicates each and three extraction controls (extracts of empty tubes), with sample injection order randomized and an injection blank (3 μl MeOH) run between each sample with the blank replaced by an injection of internal standard mix every 3rd sample as well as QC mix every 15 samples. Mass spectrometer source settings included a sheath gas flow rate of 55 (au), auxiliary gas flow of 20 (au), sweep gas flow of 2 (au), spray voltage of 3 kV and capillary temperature of 400°C.

### Metabolomic identification

Untargeted analysis was performed as described previously (https://doi.org/10.1093/plphys/kiae001). Briefly feature based molecular networking (FBMN) with MZMine 2.39 and GNPS was performed (CITE FBMN, GNPS, and MZMine). First, raw data was processed through MZMine 2.39 to identify features (unique m/z and RT pairs), with peak height and peak area exported for each feature into a table. Putative identifications were generated for each feature for which MSMS fragmentation data was collected using GNPS. The identification confidence for each feature and further information can be found in GNPS results tables (Supplemental Tables S3–S6), and the peak height of each feature in Supplemental Tables S7–S10 for both positive and negative ionization modes for both polarities.

### Statistical analysis

#### Carbon dioxide rate analysis

A Shapiro–Wilk test was used to check for normalcy of the data. As the data was found to be non-normal, a Kruskal–Wallis Test with a post-hoc Dunn Test was used to determine any significant differences in CO_2_ Rate by Community, Phenotype, and Time. Significant differences were determined by a *P*-value <.05.

#### Metagenomic analysis

To determine Species Richness, a Shapiro–Wilk test was performed to check for normality. As the data was found to be non-normal, a Kruskal–Wallis test was performed to determine significant differences in Richness by Community, Phenotype, and Time. Then a post-hoc Dunn test was performed to determine the specific differences when comparing by Community. Significant differences were determined by a *P*-value <.05. To compare the microbial diversity across samples, the data was mean-normalized using the R package DESeq2 [35]. Once normalized, the Shannon diversity and Simpson diversity indices were found using the R package “vegan” v2.6–4 [36]. An analysis of variance analysis was then used to determine the differences in diversity by community, phenotype, time, and the interaction of the factors. Significant differences were determined by a *P*-value <.05.

To analyze the taxonomy and determine relative abundance in the microbial communities, the DOE Systems Biology Knowledgebase (KBase) was used. Within KBase, the program GOTTCHA2 v2.1.7 was used to classify the taxonomy of the metagenomic reads obtained from JGI [37]. The relative abundance data output from this program was then used for statistical analyses. The dataset was first filtered to only contain the data at the genera level, then Kruskal–Wallis analysis was performed to compare by phenotype and time point. Significance was determined by *P* < .05.

For gene and gene feature analysis the DOE JGI IMG/M mentioned previously was used. The assembled genomes by phenotype (hydrophilic vs. hydrophobic) and time point (Day 45 vs. Day 89) alone as well as combined (i.e. Day 45 hydrophilic vs. Day 45 hydrophobic). These sets were compared using the Pfam database to compare protein families and their predicted functional group and significance was determined using Mann–Whitney analysis. Significance was determined by *P* < .05.

Bacterial network analyses using the genus abundance table from GOTTCHA2 were completed in RStudio version 2023.12.0 + 369 using package NetCoMi v1.1.0. Due to the sparse number of samples [36], since these were metagenomes, the “spring” (Semi-Parametric Rank-based approach for Inference in Graphical model) approach did not work. Therefore, we used SparCC (Sparse Correlations for Compositional data) approach [38]. Sparisfy associations were completed via “t-test” and adjusted for multiple testing via “adaptBH”. In total, 43 genera and 36 samples were analyzed without errors.

#### Metabolomic analysis

Metabolomic data was analyzed using the MetaboAnalyst 6.0 platform. Using this platform, the data was normalized by a pooled sampled from the background group (group PQN)

And underwent a log10 transformation and auto-scaling. The data was then analyzed using the linear model with covariate adjustment function to determine significant compounds by phenotype when adjusted for time. These results were used to pare down to the top 10 metabolites per LC/MS polarity (positive or negative) per column (HILIC or C18) which were then displayed in a heatmap to show changes in relative abundance using the R package “pheatmap” v1.0.12 [39].

## Results

### Microbial respiration did not explain soil water repellency

Microbial respiration, which is an indicator of microbial activity, was initially found to be significantly different between the hydrophilic and hydrophobic phenotypes (*P* = 2.75E-02). Upon further investigation, it was discovered that the community S17 (hydrophilic) drove this significance and when S17 values were removed, there was no significant difference in respiration between the hydrophilic and hydrophobic phenotype, indicating that microbial activity does not vary significantly between SWR phenotypes (Supplemental Fig. S1). While respiration did not vary by phenotype, it did change significantly by microbial community at each time point (*P* < 1E-4) except for Day 16 (*P* = 1.94E-01) (Supplemental Fig. S2).

### Bacterial Shannon diversity is greater in hydrophobic communities while fungal Shannon diversity increases in hydrophilic communities

**Figure 1:**
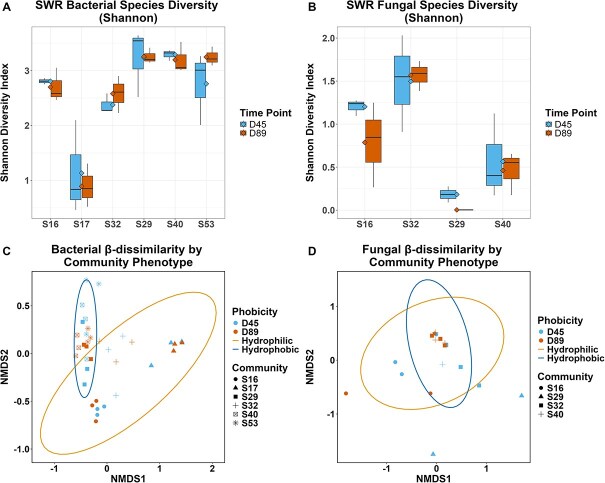
Comparing microbial community alpha and beta-diversity by hydrophilic (S16, S17, S32) and hydrophobic (S29, S40, S53) phenotypes and time points (Day 45 and Day 89). (A) Shannon diversity index of bacterial species compared between phenotype, time point, and community. (B) Shannon diversity index of fungal species compared between phenotype, time point, and community. Communities S17 and S53 are not shown in the fungal comparison (B) as neither community returned as having any fungal hits. (C) Bacterial β-diversity determined using Bray–Curtis between communities (R^2^ = 0.46, *P* = .001) and phobicity (hydrophilic = orange, hydrophobic = blue, R^2^ = 0.33, *P* = .001). Sample timepoints are differentiated by color (D45 = light blue, D89 = red), and no significance was found (R^2^ = –0.01, *P* = 0.51). (D) Fungal β-diversity determined using Bray–Curtis between communities (R^2^ = 0.35, *P* = .003) and phobicity (hydrophilic = blue, hydrophobic = orange, R^2^ = 0.04, *P* = .389). Sample timepoints are differentiated by color (D45 = light blue, D89 = red), and no significance was found (R^2^ = -0.01, *P* = .52).

Initially, significant differences in Shannon diversity were found to be significant by phenotype (*P* = 5.4E-8) and individual community (*P* = 2.36E-6). Community S17 appeared to drive this difference, due to the high abundance of *Nocardiopsis* in this community. When diversity by individual community was analyzed after excluding S17, it no longer influenced diversity (*P* = 3.15E-1). However, both before and after the exclusion of S17, bacterial diversity significantly differed by SWR phenotype with higher diversity in hydrophobic communities (*P* = 4E-4) (Fig. 1A). In contrast, fungal Shannon diversity increased in hydrophilic communities (*P* = 5E-4) (Fig. 1B). Neither bacterial nor fungal species richness differed significantly between SWR phenotypes or timepoint.

When comparing bacteria, hydrophilic communities (S16, S17, S32) had significantly more variance between communities compared to hydrophobic communities (S29, S40, S53) (*P* = 1.3E-4) (Fig. 1C). The greatest variance between samples was explained by the original soil community (R^2^ = 0.46, *P* = 1E-3) and SWR phenotype (hydrophilic or hydrophobic) explained an additional 33% of the variance between samples (*P* = 1E-3). There was no significant impact of time (*P* = 0.51, R^2^ = −0.01). When comparing fungi, the only significant source of explained variance was by original soil community (*P* = 3E-3, R^2^ = 0.35); however, only 23 out of 36 samples were used due to no fungal sequences being detected (Fig. 1D). Due to multiple communities having no fungal metagenomes detected, a network analysis could not be performed.

**Figure 2:**
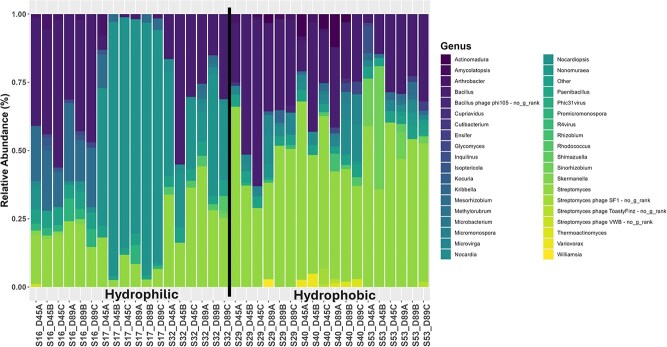
Relative abundance of bacterial genera compared between time point and phenotype. Column names are based on community ID (hydrophilic = S16, S17, S32 and hydrophobic = S29, S40, D53), timepoint (D45 = Day 45, D89 = Day 89), and replicate (a, B, C). Significant genera by time point and phobicity were determined via the Wald test with the Benjamini–Hochberg procedure. Genera denoted with ^*^ show a significant difference by phenotype, and those denoted with ^**^ show a significant difference by time point. Genera with <1% relative abundance are denoted as “other”. *P*-values for significantly different genera can be found in Supplemental Tables S11 and S12.

Microbial community composition also significantly differed by phenotype and time point with 36 bacterial genera changing in relative abundance between phenotypes and 3 changing in relative abundance between timepoints (Fig. 2, Supplemental Tables S11 and S12). By SWR phenotype, *Nocardiopsis* and *Kocuria* increased in relative abundance in the hydrophilic communities while *Cutibacterium* and *Streptomyces* increased in relative abundance in hydrophobic communities*.* The bacterial genera that changed between timepoints included *Paenarthrobacter*, which decreased over time, and *Solirubrobacter* and *Thermoactinomyces* which both increased over time. Both *Paenarthrobacter* and *Solirubrobacter* had <1% relative abundance in all samples at both timepoints. *Paenarthrobacter* was present in all communities and *Solirubrobacter* was found in S32 (hydrophilic) and S40 (hydrophobic) at D89. *Thermoactinomyces* was present in communities S16, S32 (both hydrophilic), and S40 (hydrophobic) and almost entirely in the D89 samples, however, it only reached >0.05% relative abundance in S40 (Supplemental Table S12). This suggests that while some bacterial genera are changing by time, they are not driving the development of the phenotype.

**Figure 3:**
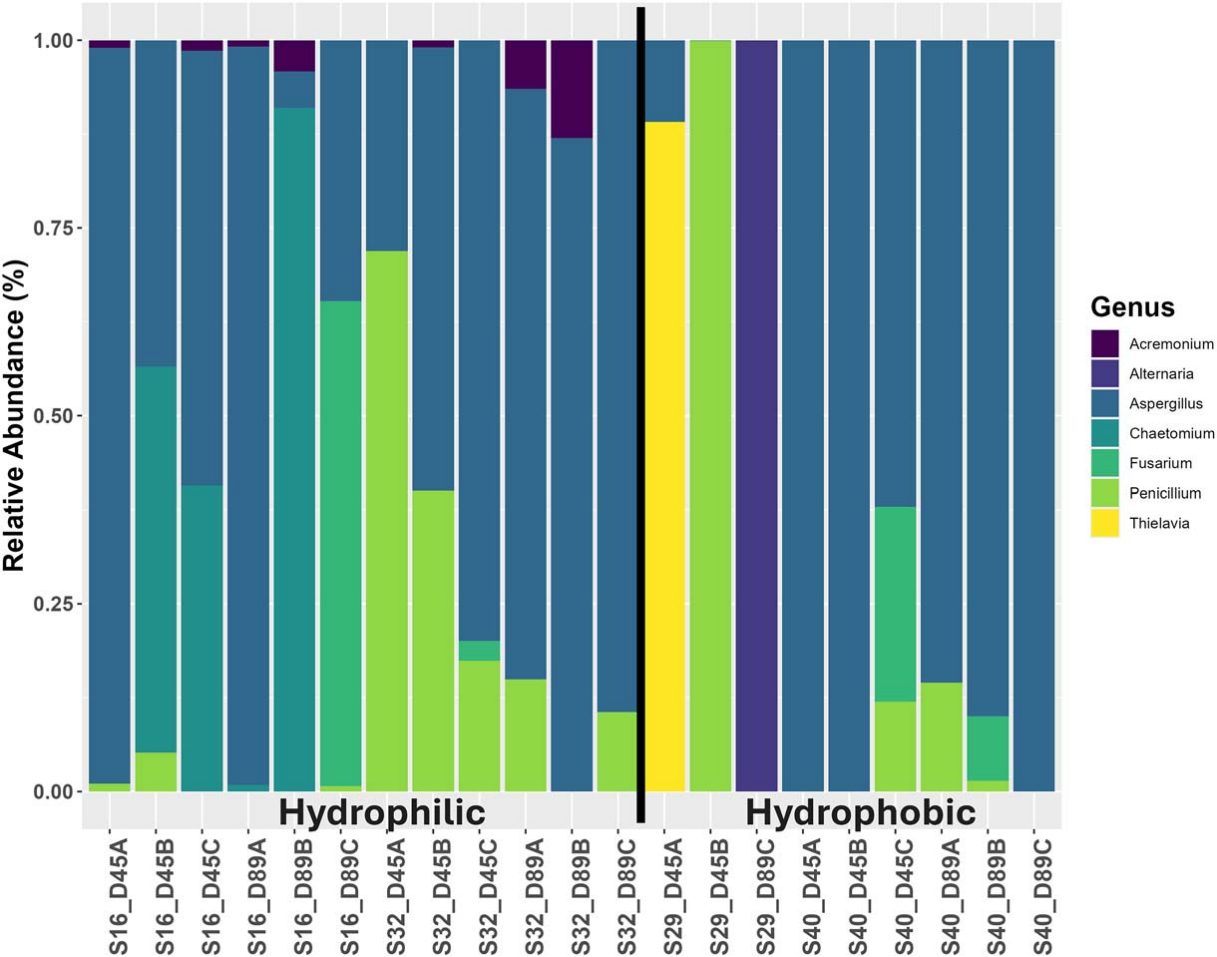
Relative abundance of fungal general compared between time point and phenotype. Column names are based on community ID (hydrophilic = S16, S32 and hydrophobic = S29, S40), timepoint (D45 = Day 45, D89 = Day 89), and replicate (A, B, C). Samples that did not return hits for fungi were excluded from the figure. Significant genera by time point and phobicity were determined via the Wald test with the Benjamini–Hochberg procedure. Genera denoted with ^*^ show a significant difference by phenotype.

When comparing fungal community abundances, no genera were significantly different between time points; however, between phenotypes, *Acremonium* was significantly more abundant in the hydrophilic communities (*P* = 7.65E-3) (Fig. 3). While the lack of fungal reads in several samples, with two communities having no reads at all, makes drawing conclusions difficult, some fungal genera are only present in communities of a given phenotype. *Acremonium* (mentioned above as being the only significant genera) is only present in hydrophilic communities, whereas *Alternaria* and *Thielavia* are only present in hydrophobic communities. This suggests that some fungal genera may play a role in repellency phenotype.

**Figure 4:**
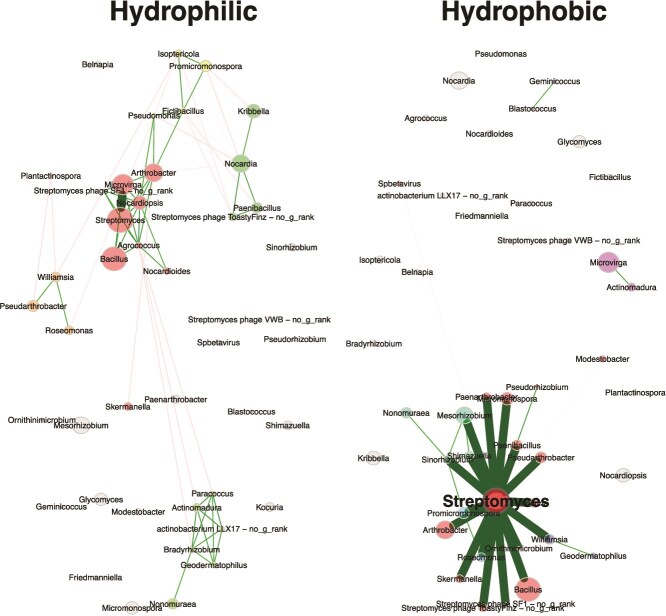
Network visualization of hydrophilic and hydrophobic bacterial genera networks based on the SparCC approach. Sparisfy associations were completed via “t-test” and adjusted for multiple testing via “adaptBH”. The node colors denote clusters and the line colors denote expected associations: positive = green and negative = red.

Community networks revealed that the hydrophilic communities are more connected than the hydrophobic communities (Fig. 4). Of note, the largest connected component in the hydrophobic network has more positive edges (86%) than the hydrophilic (70%); however, the whole network analysis shows the opposite (71% hydrophilic vs. 56% hydrophobic). The edge density in the whole network was almost double in hydrophilic (0.069) compared to hydrophobic (0.032). This all indicates that while the major connectivity hub in the hydrophobic network influences a large portion of its community, there are more interactions occurring in the hydrophilic communities overall. Modularity was also higher in hydrophilic (0.454) compared to hydrophobic (0.036). In hydrophobic communities, Streptomyces was a connectivity hub with 20 connections to other nodes, a betweenness centrality of 388, and eigenvector centrality of 0.578, indicating that it plays a major role in the interactions occurring in hydrophobic communities. The hydrophilic network has a relative largest connected component of 0.628 and a whole network clustering coefficient of 0.767.

### Hydrophobic microbial communities have an increased abundance of genes associated with defense mechanisms and secondary metabolites

Many genetic features (i.e. functional potential) significantly differed between hydrophobic and hydrophilic communities and between time points. When comparing hydrophobic and hydrophilic communities overall, 14 Pfam categories were found to be different. At single timepoints, 8 differences were observed at Day 45 and 13 at Day 89 (Table 1). Of note was an increased relative abundance of genetic features associated with carbohydrate and lipid transport and metabolism in hydrophobic communities across both timepoints (Day 45 and 89), with genes such as those for thiolase (pfams 02803, 00108) and lipases (pfams 01674, 13472, 03583) found in significantly higher abundance. Additionally, at Day 89, there was an increased relative abundance defense mechanism genetic features in hydrophobic communities including those for antimicrobial compounds and immunity proteins (e.g. pfams 03992, 14433, 14435, 03364), as well as those associated with Type VI secretion systems (pfams 05638, 05936, 05947 (Supplemental Table S13). In hydrophilic communities we observed an increased relative abundance of intracellular trafficking, secretion, and vesicular transport, such as ABC transporter genes (pfams 16 296 and 00950), flagellar genes (pfams 02154, 02119, 02108), and signaling genes (pfams 07695, 10502, 02978). This can be further linked to the synthesis and transport of osmolytes, which could be influencing the hydrophilic phenotype. Such examples include genes associated with trehalose-phosphate, an intermediate of trehalose (pfam02358), as well as genes associated with sorbitol and betaine (pfams 02028, 07355, 09338, 03612) which are other known osmolytes (Supplemental Table S13).

**Table 1:** The predicted function of genes (Pfam) categories found to significantly differ between soil water repellency phenotypes (hydrophobic or hydrophilic) and time point (Day 45 or Day 89) as determined by a Mann–Whitney test.

Predicted genetic function	Significant phobicity	Significant timepoint	Adjusted *P*-value
Intracellular trafficking, secretion, and vesicular transport	Hydrophilic	Day 45 and Day 89	0.001, 0.0002
Inorganic ion transport and metabolism	Hydrophilic	Day 45 and Day 89	0.001, 0.0006
Translation, ribosomal structure, and biogenesis	Hydrophilic	Day 45 and Day 89	0.0031, 0.0002
Cell motility	Hydrophilic	Day 45 and Day 89	0.0125, 0.0003
Replication, recombination, and repair	Hydrophilic	Day 45 and Day 89	0.0216, 0.0002
Transcription	Hydrophobic	Day 45 and Day 89	0.001, 0.0002
Carbohydrate transport and metabolism	Hydrophobic	Day 45 and Day 89	0.005, 0.003
Lipid transport and metabolism	Hydrophobic	Day 45 and Day 89	0.0125, 0.0015
Nucleotide transport and metabolism	Hydrophilic	Day 89	0.0021
Cell cycle control, cell division, and chromosome partitioning	Hydrophilic	Day 89	0.0087
Defense mechanisms	Hydrophobic	Day 89	0.0002
Signal transduction mechanisms	Hydrophobic	Day 89	0.0322
Secondary metabolite biosynthesis, transport, and catabolism	Hydrophobic	Day 89	0.0391

### Comparison of exometabolites between time point and phenotype

**Figure 5:**
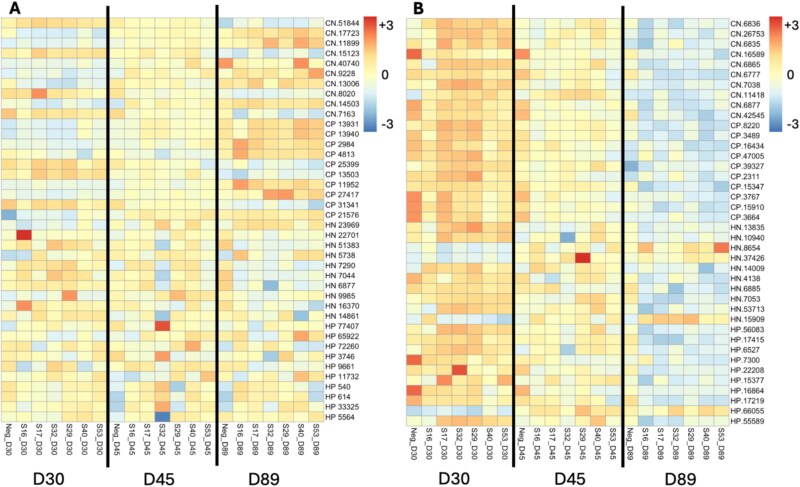
Heatmap displaying the top 10 differentially abundant metabolites per LC/MS column for a total of 40 significant metabolites associated with time point (Day 30, Day 45, and Day 89) as determined by Kruskal–Wallis analysis. (A) Top differentially abundant mass features with no known associated compound (unidentified). (B) Top differentially abundant exometabolites that have an associated database compound hit (putatively identified). The row names for metabolites consist of the LC/MS column followed by the metabolite ID. CN = C18 column-negative ion channel, CP = C18 column-positive ion channel, HN = HILIC column-negative ion channel, HP = HILIC column-positive ion channel.

The abundance of many exometabolites differed by community phenotype, with a total of 12 389 exometabolites found to be significantly different from the two LC/MS columns and two polarities (C18 Neg, C18 Pos, HILIC Neg, and HILIC Pos) combined. When controlling for time point as a covariate, 725 exometabolites (identified and unknown) were found to be significant across the four combined datasets. When comparing across timepoints, the top differentially abundant unidentified exometabolites had quite a bit of variation in the trends of their abundances, indicating that at least some significant exometabolites are being produced by the communities (Fig. 5A). In contrast, the top significantly different, putatively identified exometabolites tended to decrease in abundance over time with 36 out of 40 having the highest abundance at Day 30 (Fig. 5B).

**Figure 6:**
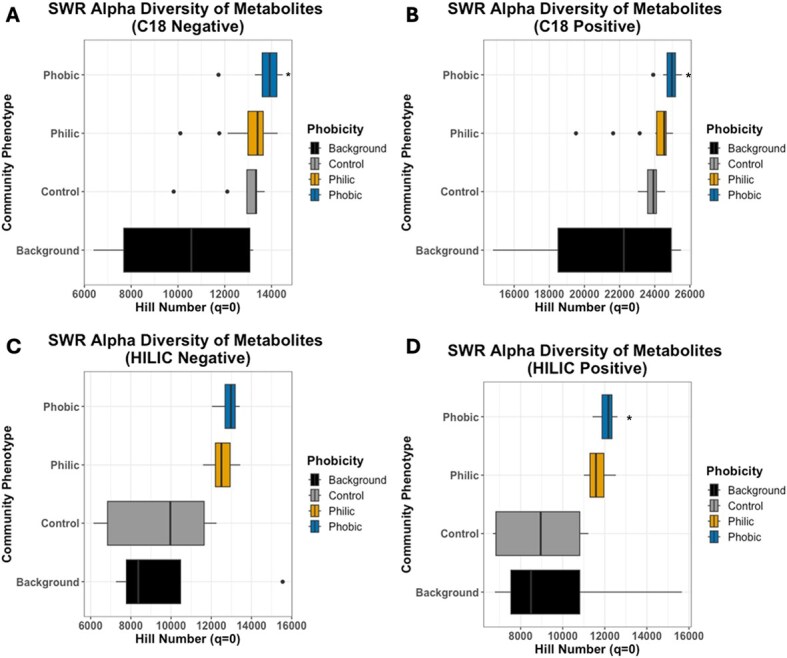
Alpha diversity of exometabolites in hydrophilic (orange) and hydrophobic (blue) communities based on calculated hill numbers for each LC/MS channel. Significant differences between SWR phenotypes were determined via Kruskal–Wallis analysis and are denoted by an *. Background samples refer to the following samples: [1] Model soil that contained no-inoculum and no litter, [2] sterile litter only that contained no inoculum or model soil, and [3] external controls for the LC/MS columns including pre- and post-run controls.

When comparing the top 40 putatively identified exometabolites between hydrophobic and hydrophilic communities, we observed that more of the exometabolites had higher abundances within hydrophobic communities (32/40 exometabolites) (Supplemental Fig. S3B). Many of the identified exometabolites found to be significant in the hydrophobic communities are linked to plant material, such as delta-hexalactone, phthalic acid derivatives, and 3-methyladipic acid, which suggests that their increase in abundance could be due to the degradation of plant litter. The presence of plant-derived antimicrobial agents (such as neoabietic acid and sulfosalicylic acid) in hydrophobic communities could also be linked to the increase of genes associated with defense mechanisms and immunity proteins (Supplemental Table S15). In the hydrophilic communities, osmolytes such as ectoine are linked to production by microorganisms like *Nocardiopsis*, previously mentioned as increasing relative abundance in hydrophilic communities (Supplemental Table S15). When further comparing the differences in metabolic profile between phenotypes, it was found that the alpha-diversity of exometabolites was significantly higher in hydrophobic communities in three out of the four LC/MS columns/polarities (the fourth not showing a significant difference) (Fig. 6A–D).

## Discussion

In this study we showed that microbial community composition interacts strongly with SWR and identified specific microbial taxa and metabolic compounds potentially driving this effect. Previous work analyzing factors contributing to SWR found a direct correlation between general microbial activity and an increase in repellency but that correlation was not found in this study [21, 24]. While variation in microbial respiration, as a proxy for activity, was seen between communities, it was not found to differ by SWR phenotype, indicating that this phenomenon is not the driver of the observed repellency phenotype (Supplemental Fig. S1).

Consistent with previous work [13, 40], the presence of differing microbial communities was found to drive the repellency status of otherwise identical synthetic soils (Figs 1, 4, and 5). Overall, 33% of variance in bacterial community composition was explained by phobicity status (hydrophilic or hydrophobic) while phobicity did not explain the variance in the fungal community composition, rather 35% of the variance in fungal community composition was explained by the original soil inoculum (Fig. 1C and D). Time also did not explain the variance found in either bacterial or fungal community composition. This is most likely because down selected communities for further metagenomic and metabolomic study were chosen for their consistent display of the same phenotype over time. Several of the initial 15 communities underwent a change in phenotype over the course of the experiment (hydrophilic to hydrophobic or the reverse) and while these were not selected for further analysis in this study, future research should focus on the phenomena that causes this switch (Supplemental Table S1). Within the hydrophilic communities, community S17 was primarily composed of the bacterial genus *Nocardiopsis*, known for its production of biosurfactants and its desiccation-resistant behavior [41]. While communities S16 and S32 also contained *Nocardiopsis*, the abundances were nowhere near the level in S17 (Fig. 2, Supplemental Table S11). The contribution of *Nocardiopsis* to the hydrophilic phenotype is supported by our metabolomic data, as the metabolite with the highest significance in the hydrophilic communities is ectoine, a compound known to be produced by *Nocardiopsis* species [42, 43]. Another genus contributing to the hydrophilic communities is *Kocuria*, which is predominantly found in S16 and S32 with lower numbers in S17 and is known to produce biosurfactants as well [44, 45].

In the case of the hydrophobic communities three genera stood out, *Streptomyces*, *Kribbella*, and *Cutibacterium*. *Streptomyces* are extremely common in soil, and we observed them in all our communities; however, the hydrophobic communities had a significantly higher abundance. Previous research has found that *Streptomyces* secrete a set of hydrophobic proteins, ChpA-H, involved in the formation of its aerial hyphae [46]. The genes *chpA-C*, encoding the proteins ChpA-C, were found to be significantly associated with the hydrophobic phenotype in the metagenomic analysis (Supplemental Table S13), indicating a means through which *Streptomyces* could be influencing the hydrophobic phenotype. These findings combined with the fact that *Streptomyces* is such a significant connectivity hub with connections to 20 other nodes (Fig. 4) suggests that it is a major influencer in the microbial communities of hydrophobic soils. *Kribbella* has been previously isolated from soils in arid climates and observed to increase abundance under water stress conditions [47, 48]. Both *Streptomyces* and *Kribbella* have also been indicated as having high anti-fungal activity [49], providing a potential link to the high abundance of genes associated with defense mechanisms, as well as another potential explanation to the lower fungal diversity and abundance associated with the hydrophobic communities. Finally, *Cutibacterium* is only found in the hydrophobic communities. Previous research done on the human skin microbiome has found instances in which *Cutibacterium* produces hydrophobic compounds to form a lid over its active site and, as this same *Cutibacterium* has been found associated with grapevines, it is possible that this could also cause an impact on the repellency status of soil [50, 51]. Going against previously documented findings, a significantly higher relative abundance of fungal genera was associated with the hydrophilic phenotype, despite being entirely absent from community S17 (Fig. 3). Fungi are known to be generally hydrophobic organisms with many studies linking their presence to increased SWR [17]. However, other studies have found that, due to their hydrophobic nature, fungi preferentially consume hydrophobic compounds in the soil, and this consumption has led to increased philicity of soil [17, 52]. This suggests that the production and consumption of specific compounds may be the main driver of SWR. This hypothesis is supported by the metabolomic data analysis performed (Figs 5 and 6, Supplemental Fig. S3, Supplemental Tables S14 and S15). Some compounds associated with either the hydrophilic or hydrophobic phenotype have been found to have either hydrophilic or hydrophobic properties themselves, such as ectoine, a known osmolyte and hydrophilic compound [53], or neoabietic acid, a highly hydrophobic resin acid [54]. Further research into the top 40 known metabolites showed that many of those associated with hydrophobic communities are linked to plant material and production, suggesting that their presence is due to the degradation of plant litter (Supplemental Table S15). It could be that the hydrophobic communities have a differing level of decomposition ability than the hydrophilic communities, which could be contributing to the hydrophobic phenotype. Additionally, as the hydrophobic microbial communities are less connected (Fig. 4), the products of plant decomposition could be accumulating whereas the hydrophilic communities may be able to consume these products. It is also possible that some of the top unknown metabolites are driving the observed phenotype as it is likely that many of these unknown metabolites are of microbial origin and are entirely unstudied.

Overall, we identified several bacterial genera that may be influencing SWR including *Nocardiopsis*, *Kocuria*, *Streptomyces*, and *Cutibacterium*, and key characteristics of these genera that support these findings. Through metagenomic and metabolomic analyses we were also able to identify potential compounds, some linked to the significant genera, that may directly affect the phobicity of the soil. Finally, our evidence suggests that fungi, previously thought to cause hydrophobicity, may actually contribute to hydrophilicity through their consumption of hydrophobic compounds. Further understanding the impact of these metabolic compounds on soils and microbial production/consumption of these compounds is necessary to determine if bioaugmentation of soils using specific microorganisms and/or metabolites could improve SWR.

## Supplementary Material

SWR_Supplemental_Figures_ycaf084

SWR_Supplemental_Tables_ycaf084

## Data Availability

The work (proposal: https://doi.org/10.46936/10.25585/60001346) conducted by the U.S. Department of Energy Joint Genome Institute (https://ror.org/04xm1d337), a DOE Office of Science User Facility, is supported by the Office of Science of the U.S. Department of Energy operated under Contract No. DE-AC02-05CH11231. The massive accession number for the raw metabolomic data is MSV000094090. The metagenome sequencing raw data were deposited in the JGI IMG/MER database and the metagenome IDs are provided in Supplemental Table S2. Raw metabolomics data is available at MassIVE under accession number: MSV000094090. (doi:10.46936/10.25585/60001346). The GNPS FBMN jobs are available at the following links: https://gnps.ucsd.edu/ProteoSAFe/status.jsp?task=c9740d2c968e4475a9ffa459c357fb39, https://gnps.ucsd.edu/ProteoSAFe/status.jsp?task=8b3f99b5419c4465ac2458b6e639d3db, https://gnps.ucsd.edu/ProteoSAFe/status.jsp?task=eafc227ac8d14f90ae7412558f24fbdd, and https://gnps.ucsd.edu/ProteoSAFe/status.jsp?task=a2f469fcef804509b16bdb8fc7500e11.
