# Supplementary material for: Bacterial and fungal composition and exometabolites control the development and persistence of soil water repellency
Source: ISME Commun. 2025 May 20;5(1):ycaf084. doi: 10.1093/ismeco/ycaf084 (PMC12143472; doi:10.1093/ismeco/ycaf084)
Supplement: SWR_Supplemental_Figures_ycaf084 [file swr_supplemental_figures_ycaf084.docx]

**
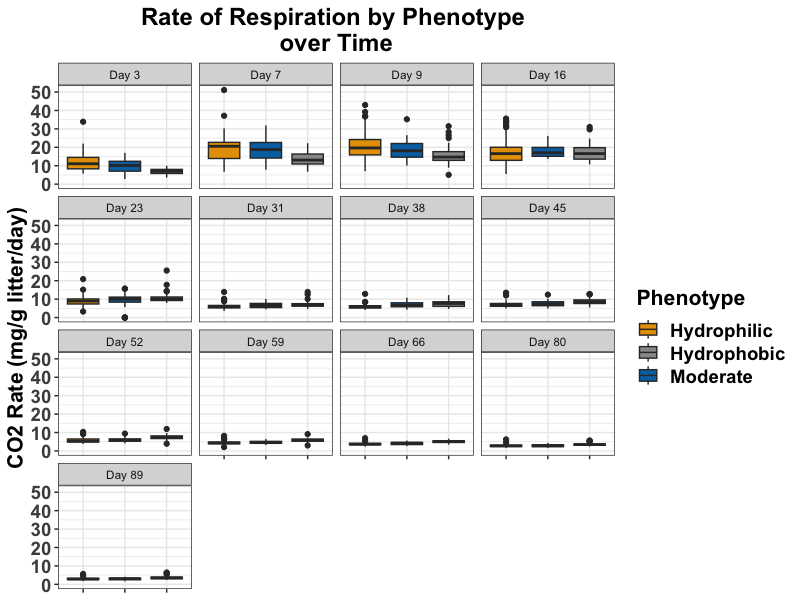
**

**Supplemental Figure 1**: **Comparing Microbial Respiration Rates over time by community final hydrophilic (S16, S17, S32, S35, S49, S76, S89), hydrophobic (S20, S29, S40, S53), and moderate (S24, S39, S47, S48) phenotypes.** Respiration rates of each microbial community were compared using Kruskal-Wallis analysis and a post-hoc Dunn Test. Statistics indicate that significant differences observed between phenotypes is driven by the community S17.


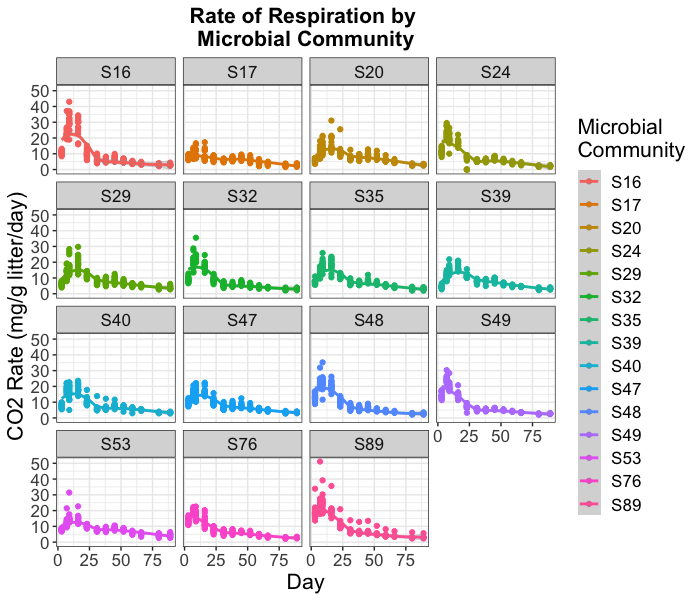


**Supplemental Figure 2: Respiration Rate associated with microbial community phenotype by time point.** Microbial community respiration rates grouped by phenotype at each time point were analyzed using a Kruskal-Wallis test and a post-hoc Dunn test. There were significant differences between hydrophilic and hydrophobic phenotypes at all time points except for Day 16.


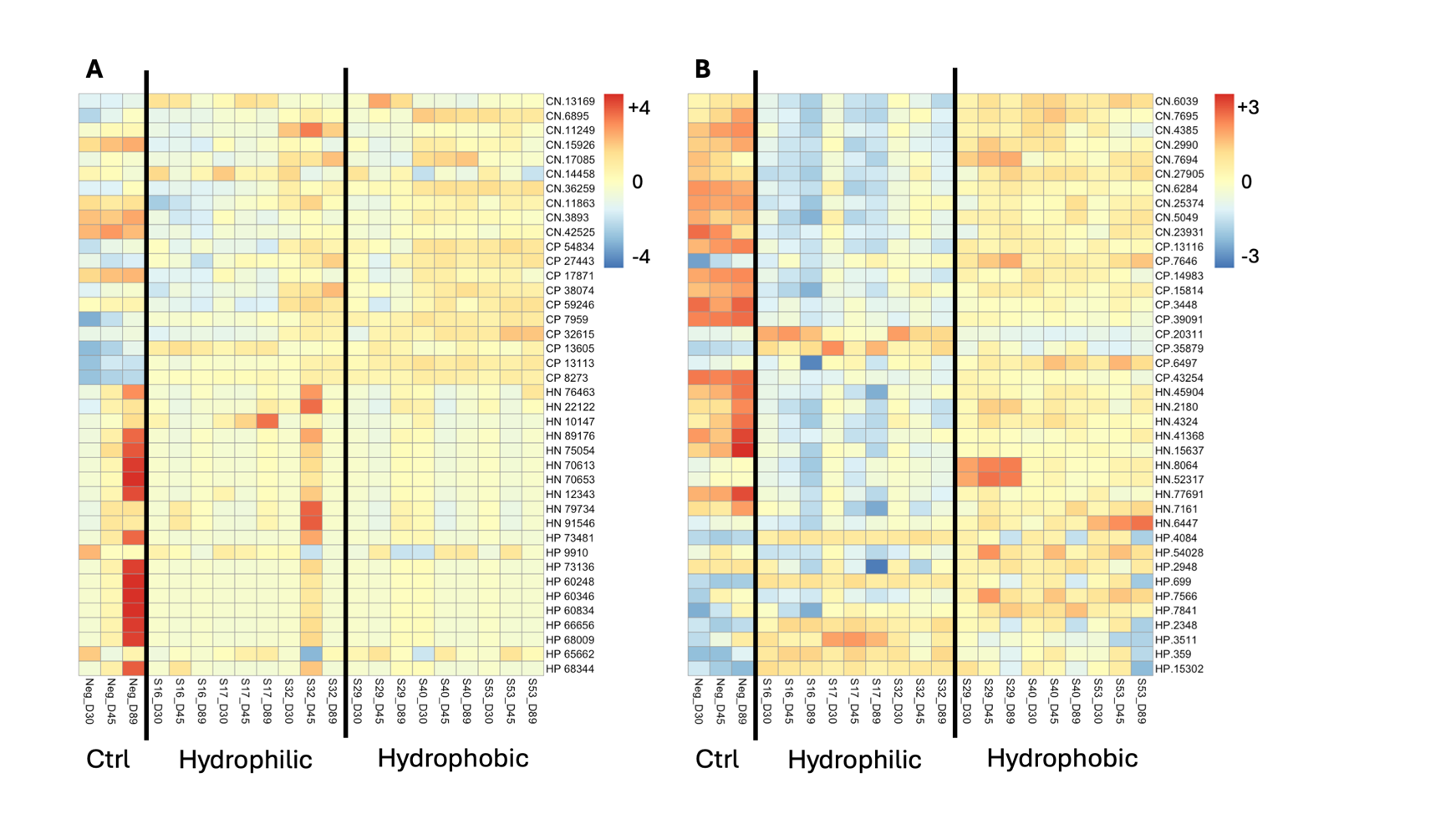


**Supplemental Figure 3**: Heatmap displaying the top 10 metabolites per LC/MS channel associated with phenotype (hydrophilic and hydrophobic) as determined by Kruskal-Wallis analysis. A) Top metabolites that do not have an associated top scoring compound (unknowns). B) Top metabolites that have an associated top scoring compound (potential identity).
